# Living Donor Kidney Transplant in Recipients With Glomerulonephritis: Donor Recipient Biologic Relationship and Allograft Outcomes

**DOI:** 10.3389/ti.2023.11068

**Published:** 2023-05-05

**Authors:** Rasha El-Rifai, Adam Bregman, Nattawat Klomjit, Richard Spong, Scott Jackson, Patrick H. Nachman, Samy Riad

**Affiliations:** ^1^ Division of Renal Diseases and Hypertension, Department of Medicine, University of Minnesota, Minneapolis, MN, United States; ^2^ Complex Care Analytics, MHealth Fairview, Minneapolis, MN, United States; ^3^ Division of Nephrology and Hypertension, Mayo Clinic, Rochester, MN, United States

**Keywords:** graft survival, long term outcomes, disease recurrence, living related donor, glomerulonephritis

## Abstract

Using the Scientific Registry of Transplant Recipients, we examined the association between donor-recipient biologic relationship and long-term recipient and allograft survival among glomerulonephritis (GN) patients. Four GN types were studied: membranous nephropathy, IgA, lupus-associated nephritis, and focal segmental glomerulosclerosis (FSGS). We identified all adult primary living-donor recipients between 2000 and 2018 (*n* = 19,668): related (*n* = 10,437); unrelated (*n* = 9,231). Kaplan-Meier curves were generated for the recipient, death-censored graft survival and death with functioning graft through ten years post-transplant. Multivariable Cox proportional hazard models were used to examine the association between the donor-recipient relationship and outcomes of interest. There was an increased risk for acute rejection by 12 months post-transplant among the unrelated compared to the related group in IgA (10.1% vs. 6.5%, p<0.001), FSGS (12.1% vs. 10%, *p*-0.016), and lupus nephritis (11.8% vs. 9.2%; *p*-0.049). The biological donor-recipient relationship was not associated with a worse recipient or graft survival or death with functioning graft in the multivariable models. These findings are consistent with the known benefits of living-related-donor kidney transplants and counter the reports of the potential adverse impact of the donor-recipient biologic relationship on allograft outcomes.

## Graphical Abstract

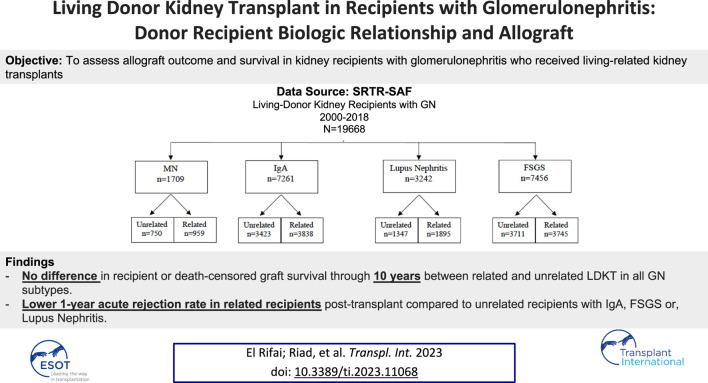

## Introduction

Glomerulonephritis (GN) is among the major causes of ESKD accounting for about 30% of kidney transplants in the United States (USRDS report 2015) (1). Common glomerulonephritides such as membranous nephropathy (MN), IgA nephropathy, and focal segmental glomerulosclerosis (FSGS) can recur at variable rates ranging between 35% and 50% at 5 years post-transplant (2). Recurrent disease is the third most common cause of allograft loss after chronic rejection and death with a functioning graft (3). About 15% of death censored graft failures are attributed to recurrent GN’s though this may be an underestimate of the true incidence especially with the challenges distinguishing between *de novo* and recurrent GN (2).

Controversies exist about the association of donor recipient biologic relationship with allograft outcomes for recipients with ESKD due to glomerulonephritis. An earlier study by Choy et al in 2006 (4) suggested that biologic relationship between donor and recipient was not associated with an increased risk of GN recurrence after kidney transplant. However, a more recent study by Kennard et al in 2017 (5) including 7,236 patients from ANZDATA transplant registry data showed a significantly higher 10-year risk of disease recurrence among living related compared to living unrelated donor kidney transplant recipients (16.2% vs. 10.3%, respectively). This effect was observed among recipients with IgA nephropathy and FSGS.

A more recent study by Husain et al examined the association between donor-recipient biologic relationship and allograft survival after living donor kidney transplant (1). The study included more than 72,980 living donor kidney transplants in the US between 2000 and 2014, 59% (43,147) were biologically related. After adjustment for multiple donor and recipient characteristics including HLA matching, donor recipient biologic relationship was associated with a slightly higher risk for death censored graft failure. However, the study did not focus on recipients with ESKD due to GN.

Data on strong familial clustering of certain GNs (6) are accumulating. Accordingly, we sought to focus our analysis on the association between donor-recipient relationship in GN recipients. We studied the four most common GN disorders: membranous nephropathy (MN), IgA nephropathy, lupus nephritis (LN), and focal segmental glomerulosclerosis (FSGS).

## Materials and Methods

### Data Source

This study used data from the Scientific Registry of Transplant Recipients (SRTR). The SRTR data system includes data on all donor, wait-listed candidates, and transplant recipients in the US, submitted by the members of the Organ Procurement and Transplantation Network (OPTN). The Health Resources and Services Administration (HRSA), U.S. Department of Health and Human Services provides oversight to the activities of the OPTN and SRTR contractors.

### Study Population

**FIGURE 1:**
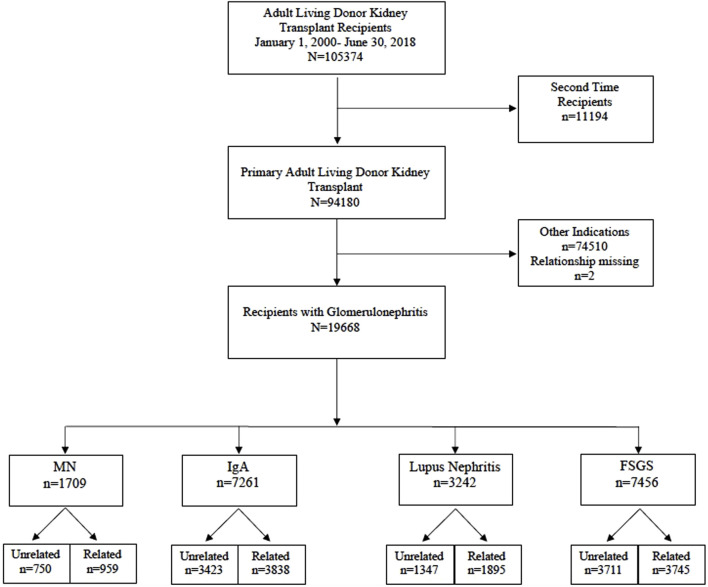
Flow Diagram of the study cohort (2000–2018).

We analyzed the scientific registry of transplant recipients (SRTR) standard analysis file for all primary kidney transplants in recipients with GN between 1 January 2000 and 30 June 2018. The 4 GN subtypes we studied included membranous nephropathy (MN), IgA nephropathy, lupus nephritis (LN), and FSGS. We excluded recipients younger than 18 years or those who received a previous kidney transplant. Our final cohort consisted of 19,668 kidney transplant recipients (Figure 1). Donor recipient pairs were classified as related if any biological relationship was reported: parent, child, sibling, or twin (*n* = 10,437) or unrelated otherwise (*n* = 9,231). Recipients were categorized according to the type of GN: MN (*n* = 1709), IgA (*n* = 7,261), LN (*n* = 3,242), and FSGS (*n* = 7,456).

### Outcomes of Interest

The primary outcomes were 10-year recipient survival and death censored kidney graft survival (DCGS). Secondary outcomes were rejection and recipient eGFR by CKD-EPI 2021 (7) at 1 year post transplant. We also assessed the cause of graft loss among the two recipient groups across the different GN types. However, the high degree of missingness did not allow for meaningful statistical analysis.

### Statistical Analysis

Univariate comparisons for recipient and death-censored graft survival outcomes were performed using Kaplan-Meier curves with log-rank tests. All survival analyses were censored at 10 years post-transplant. Multivariable Cox proportional hazard model was used to examine the association between donor-recipient relation and outcomes of interest. Each GN subtype was analyzed separately. The recipient survival, death censored graft survival (DCGS) and death with functioning graft models were adjusted for pertinent recipient and donor factors. Recipient factors included: age, sex, ethnicity, years on dialysis, preemptive status, HLA mismatch, cross match, induction, maintenance immunosuppression, and transplant year. Donor factors included donor age, sex, ethnicity, BMI, HTN, and eGFR. Covariates were included based on clinical relevance to avoid unnecessary investigator biases. Proportional hazards assumption was assessed using Schoenfeld residuals (cox.zph in the R survival package). All analyses were performed in R (ver. 4.0.2).

## Results

### Baseline Characteristics

The study included 19,668 adult primary living donor kidney transplants in recipients with GNs between 1 January 2000 and 30 June 2018; 53% (*n* = 10,437) included related donor-recipient pairs, and 47% (*n* = 9,231) unrelated pairs. Four GN groups were included in the study cohort: membranous nephropathy (MN) of which *n* = 959 recipients had a living related donor kidney transplant (LRKT) and *n* = 750 recipients had living unrelated donor kidney transplant (LUKT); IgA nephropathy recipients of LRDKT were *n* = 3,838 and LURDKT *n* = 3,423. Additionally, lupus nephritis (LN) recipients of LRDKT were *n* = 1895 and LURDKT *n* = 1,347, and FSGS recipients of LRDKT were *n* = 3,745 and LURDKT *n* = 3,711 (Figure 1). Detailed description of the cohort is included in the baseline characteristics Tables 1, 2 and can be summarized as follows: across the study population, LRDKT recipient-donor pairs tended to be slightly younger, had fewer HLA mismatches and shorter dialysis vintage.

**TABLE 1:** Baseline Recipient and Donor characteristics in Membranous and IgA N (%) or Mean [SD].

	Membranous		IgA	
	Unrelated *n* = 750	Related *n* = 959	Unrelated *n* = 3,423	Related *n* = 3,838
Recipient Characteristics				
Recipient Age	46.72 [13.8]	43.64 [15.4]**	42.99 [11.6]	40.89 [12.8]
Recipient Male	497 (66.3)	600 (62.6)	2,430 (71.0)	2,667 (69.5)
Race				
White	623 (83.1)	803 (83.7)	2,824 (82.5)	3,155 (82.2)
Black	77 (10.3)	108 (11.3)	113 (3.3)	138 (3.6)
Other	50 (6.7)	48 (5.0)	486 (14.2)	545 (14.2)
BMI(Kg/m^2^)	27.07 [5.3]	26.49 [5.6]*	27.31 [5.2]	26.74 [5.2]
HLA-MM	4.37 [1.3]	2.36 [1.4]**	4.35 [1.25]	2.22 [1.49]
PRA				
0%–20%	686 (91.8)	888 (93.9)	3,055 (90.1)	3,478 (92.2)
20%–80%	51 (6.8)	45 (4.8)	272 (8.0)	250 (6.6)
80%–100%	10 (1.3)	13 (1.4)	63 (1.9)	46 (1.2)
XM Positive	41 (5.7)	34 (3.6)	70 (2.1)	92 (2.5)
Induction	Overall *p* < 0.001		Overall *p* < 0.001	
Depletional	350 (53.4)	358 (43.8)	1864 (61.0)	1,580 (48.3)
Non-Depletional	217 (33.1)	298 (36.4)	863 (28.2)	1,159 (35.5)
Mixed	24 (3.7)	11 (1.3)	79 (2.6)	64 (2.0)
None	65 (9.9)	151 (18.5)	252 (8.2)	465 (14.2)
CNI Maintenance			Overall *p* < 0.001	
Cyclosporine	98 (13.3)	164 (17.4)	317 (9.4)	520 (13.8)
None	40 (5.4)	50 (5.3)	143 (4.2)	190 (5.0)
Tacrolimus	600 (81.3)	731 (77.4)	2,915 (86.4)	3,068 (81.2)
Steroid	522 (70.7)	683 (72.2)	2,280 (67.4)	2,619 (69.2)
Mycophenolate	661 (89.6)	831 (87.8)	3,140 (92.9)	3,421 (90.4)**
mTOR	15 (2.0)	20 (2.1)	49 (1.4)	71 (1.9)
Pre-emptive Transplant	245 (31.8)	354 (34.4)	1,191 (34.3)	1,474 (37.9)**
Dialysis Vintage (months)	21.9[22.6]	19.2[20.7]*	18.5[19.6]	15.3 [18.7]**
Donor Characteristics				
Age	42.73 [11.5]	40.36 [11.5]**	41.55 [10.8]	40.86 [11.6]*
Male	285 (38.0)	429 (44.7)*	1,309 (38.2)	1703 (44.4)**
Race	Overall *p* = 0.006		Overall *p* < 0.001	
White	664 (88.5)	803 (83.7)	3,004 (87.8)	3,171 (82.6)
Black	50 (6.7)	106 (11.1)	118 (3.4)	132 (3.4)
Other	36 (4.8)	50 (5.2)	301 (8.8)	535 (13.9)
BMI (Kg/m^2^)	26.45 [4.15]	26.65 [4.59]	26.52 [4.77]	29.35 [101.8]
Hypertension	20 (3.6)	14 (2.4)	67 (2.3)	71 (2.5)
CKD-EPI 2021 eGFR (mL/min/1.73m^2^)	96.7 [16.2]	99.0 [17.4]*	97.6 [16.45]	98.06 [16.72]

* Significance level <0.05; ** Significance level <0.001; BMI: body mass index; HLA-MM: human leukocytic antigen mismatch; PRA: panel reactive antibody; XM: crossmatch; CNI: calcineurin; mTOR: mammalian target of rapamycin; CKD-EPI 2021: chronic kidney disease epidemiology collaboration; eGFR: estimated glomerular filtration rate.

**TABLE 2:** Baseline recipient and donor characteristics in SLE and FSGS N (%) or Mean [SD].

	SLE		FSGS	
	Unrelated *n* = 1,347	Related *n* = 1,895	Unrelated *n* = 3,711	Related *n* = 3,745
Recipient Age	39.3 [11.1]	37.2 [12.0]**	45.1 [13.5]	42.1 [15.0]**
Recipient Male	282 (20.9)	337 (17.8)*	2,421 (65.2)	2,389 (63.8)
Race				
White	823 (61.1)	1,176 (62.1)	2,693 (72.6)	2,805 (74.9)
Black	398 (29.5)	535 (28.2)	851 (22.9)	795 (21.2)
Other	126 (9.4)	184 (9.7)	167 (4.5)	145 (3.9)
BMI (Kg/m^2^)	25.1 [5.2]	24.6 [5.5]*	28.4 [5.6]	27.5 [5.8]**
HLA-MM	4.4 [1.28]	2.2 [1.48]**	4.4 [1.22]	2.3 [1.42]**
PRA	Overall *p* = 0.004		Overall *p* < 0.001	
0%–20%	1,087 (81.5)	1,611 (85.7)	3,269 (88.9)	3,391 (92.1)
20%–80%	169 (12.7)	194 (10.3)	338 (9.2)	235 (6.4)
80%–100%	77 (5.8)	74 (3.9)	69 (1.9)	56 (1.5)
XM Positive	115 (9.0)	176 (9.6)	103 (2.9)	109 (3.0)
Induction	Overall *p* < 0.001		Overall *p* < 0.001	
Depletional	738 (62.6)	761 (47.8)	1935 (60.8)	1,517 (48.8)
Non-Depletional	313 (26.5)	550 (34.5)	933 (29.3)	1,083 (34.8)
Mixed	41 (3.5)	55 (3.5)	73 (2.3)	69 (2.2)
None	87 (7.4)	227 (14.2)	242 (7.6)	441 (14.2)
CNI Maintenance	Overall *p* < 0.001		Overall *p* < 0.001	
Cyclosporine	109 (8.3)	234 (12.6)	374 (10.3)	519 (14.2)
None	57 (4.3)	90 (4.8)	131 (3.6)	176 (4.8)
Tacrolimus	1,155 (87.4)	1,533 (82.6)	3,125 (86.1)	2,970 (81.0)
Steroid	1,054 (79.6)	1,485 (79.5)	2,452 (67.3)	2,558 (69.6)*
Mycophenolate	1,194 (90.2)	1,678 (89.8)	3,347 (91.8)	3,262 (88.8)**
mTOR	35 (2.6)	37 (2.0)	68 (1.9)	65 (1.8)
Pre-emptive Transplant	285 (20.4)	426 (28.3)	1,331 (35.3)	1,435 (37.6)*
Dialysis Vintage (months)	28.8 [28.2]	23.04 [22.2]**	22.2 [23.6]	17.8 [22.8]**
Donor Characteristics				
Age	40.11 (10.82)	40.03 (11.57)	42.32 (11.56)	40.30 (11.52) **
Male	593 (44.0)	838 (44.2)	1,404 (37.8)	1,614 (43.1) **
Race	Overall *p* < 0.001		Overall *p* < 0.001	
White	1,013 (75.2)	1,183 (62.4)	3,050 (82.2)	2,804 (74.9)
Black	272 (20.2)	526 (27.8)	533 (14.4)	788 (21.0)
Other	62 (4.6)	186 (9.8)	128 (3.4)	153 (4.1)
BMI (Kg/m^2^)	208.6 (6490.7)	27.10 (5.2)	27.06 (9.3)	29.61 (100.2)
Hypertension	29 (2.7)	36 (2.7)	87 (2.8)	95 (3.5)
CKD-EPI 2021 eGFR (mL/min/1.73 m^2^)	99.75 (17.4)	100.87 (17.5)	98.26 (17)	99.54 (17.5)*

* Significance level <0.05; ** Significance level <0.001; BMI, body mass index; HLA-MM, human leukocytic antigen mismatch; PRA, panel reactive antibody; XM, crossmatch; CNI, calcineurin; mTOR, mammalian target of rapamycin; CKD-EPI 2021, chronic kidney disease epidemiology collaboration; eGFR, estimated glomerular filtration rate.

### Primary and Secondary Outcomes by GN Type

#### Membranous

**FIGURE 2:**
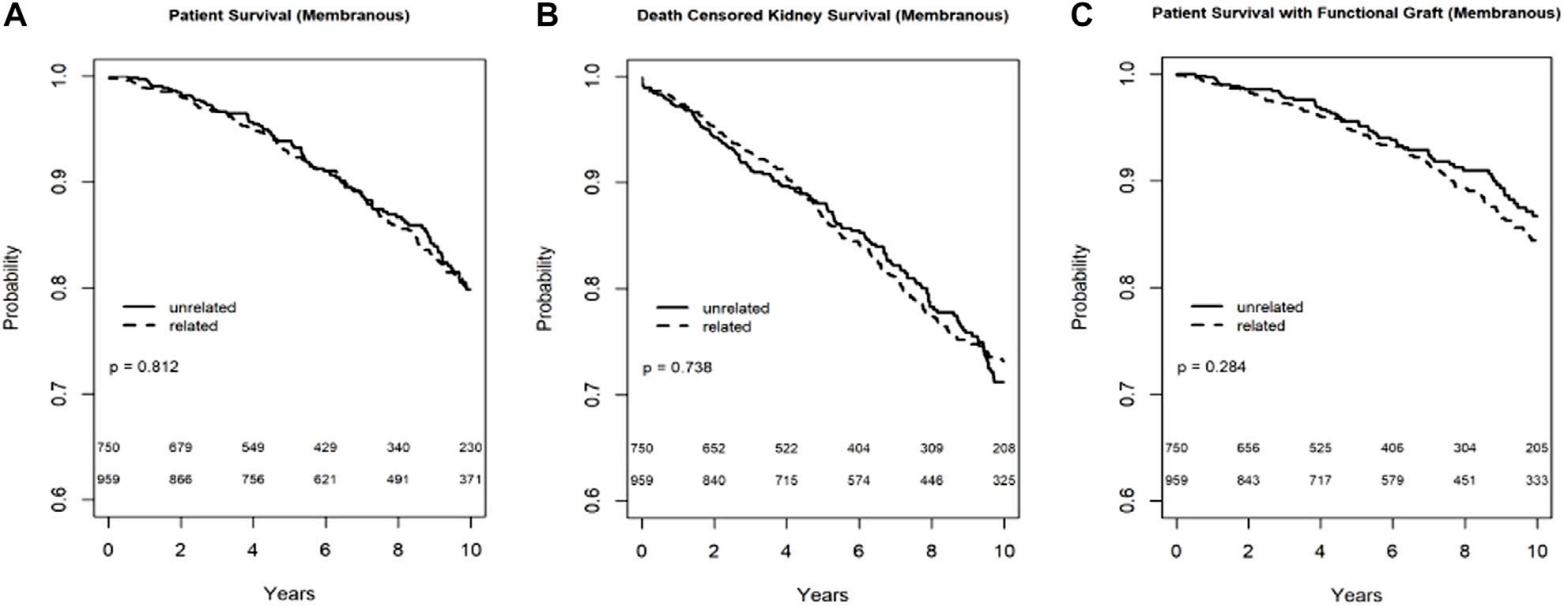
Recipient survival **(A)**, death censored graft survival **(B)** and death with functioning graft **(C)** in MN patients by biologic relationship.

In the Kaplan-Meier analysis for recipient survival in those with membranous nephropathy, donor-recipient biologic relationship was not a predictor of recipient survival (log-rank, *p* = 0.81). Similarly, in the Kaplan-Meier analyses for death-censored graft survival and death with functioning graft in recipients with membranous nephropathy, donor-recipient biologic relationship was not associated with worse graft survival (log-rank *p* = 0.82) or death with functioning graft (log-rank *p* = 0.28) (Figures 2A–C). In the multivariable models, donor recipient relationship was not associated with neither recipient [HR 1.04, 95%C.I. (0.73, 1.49), *p* = 0.82] nor death-censored graft survival [HR 1.18, 95%C.I. (0.89, 1.57), *p* = 0.24] or death with functioning graft [HR 1.12, 95%C.I. (0.72, 1.73), *p* = 0.62] (Table 3). The 1-year rejection rate and eGFR were similar among recipients with membranous nephropathy irrespective of donor type (Table 4).

**TABLE 3:** Multivariable Cox Proportional Hazard Model for Recipient and Death Censored Graft Survival by GN type.

	Recipient survival HR, 95% C.I., *p*-value	Death censored graft survival HR, 95% C.I., *p*-value	Death with functioning graft HR, 95% C.I., *p*-value
Membranous	1.04, (0.73, 1.48), *p* = 0.81	1.18, (0.89, 1.57), *p* = 0.31	1.12, (0.72, 1.73), *p* = 0.62
IgA Nephropathy	1.02, (0.77, 1.36), *p* = 0.88	1.01, (0.84, 1.21), *p* = 0.96	0.97, (0.69, 1.37), *p* = 0.86
Lupus Nephritis	0.87, (0.66, 1.16), *p* = 0.34	0.87, (0.70, 1.07), *p* = −0.18	0.84, (0.58, 1.23), *p* = 0.38
FSGS	1.06, (0.87, 1.29), *p* = 0.57	1.09, (0.93, 1.28), *p* = 0.29	0.92, (0.73, 1.17), *p* = 0.30
All	1.00, (0.88, 1.14), *p* = 0.97	1.02, (0.93, 1.13), *p* = 0.62	0.94, (0.80, 1.11), *p* = 0.46

C.I., confidence interval; FSGS, focal segmental glomerulosclerosis. Models are adjusted for recipient and donor age, gender, race, recipient years on dialysis, preemptive status, HLA, cross match, transplant year; donor HTN, eGFR, BMI. HRs are for biologically related donor-recipient pair (reference group, unrelated donor-recipient pair).

**TABLE 4:** Secondary outcomes among Related vs. Unrelated kidney transplant recipients with different GN types N (%), Mean [SD].

	Membranous		IgA	
	Unrelated *n* = 750	Related *n* = 959	Unrelated *n* = 3,423	Related *n* = 3,838
6-Month Rejection	46 (8.3)	35 (5.7)	228 (7.8)	129 (4.5)**
12-Month Rejection	59 (10.9)	50 (8.5)	286 (10.1)	181 (6.5)**
12-Month CKD-EPI 2021 eGFR (mL/min/1.73 m^2^)	58.3 [19.2]	59.5 [18.9]	60.8 [17.3]	61.2 [17.7]

	SLE		FSGS	
	Unrelated *n* = 1,347	Related *n* = 1,895	Unrelated *n* = 3,711	Related *n* = 3,745
6-Month Rejection	86 (8.1)	84 (6.1)	264 (8.8)	200 (7.3)
12-Month Rejection	122 (11.8)	122 (9.2)*	353 (12.1)	263 (10.0)*
12-Month CKD-EPI 2021 eGFR (mL/min/1.73 m^2^)	63.9 [21.4]	65.7 [21.7]*	59.2 [18.94]	60.1 [18.9]*

* Significance level <0.05; ** Significance level <0.001.

#### IgA Nephropathy

**FIGURE 3:**
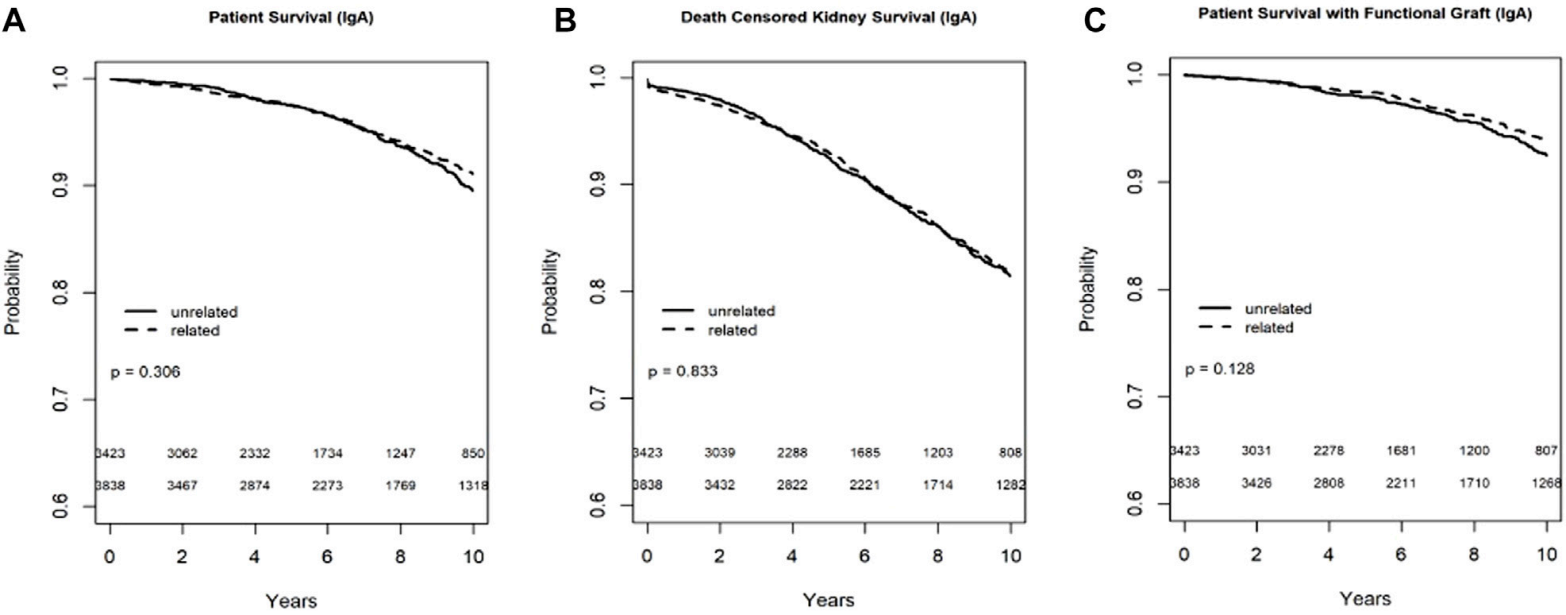
Recipient survival **(A)**, death censored graft survival **(B)** and death with functioning graft **(C)** in IgA patients by biologic relationship.

In the Kaplan-Meier analysis for recipient survival in those with IgA nephropathy, donor-recipient biologic relationship was not a predictor of recipient survival (log-rank, *p* = 0.31). Similarly, in the Kaplan-Meier survival for death-censored graft survival and death with functioning graft in recipients with IgA nephropathy, donor-recipient biologic relationship was not associated with worse graft survival (Log-rank, *p* = 0.83) or death with functioning graft (log-rank *p* = 0.13) (Figures 3A–C). In the multivariable models, donor recipient relationship was not associated with neither recipient [HR 1.02, 95%C.I. (0.77, 1.36), *p* = 0.88], death-censored graft survival [HR 1.01, 95%C.I. (0.84, 1.21), *p* = 0.96] or death with functioning graft [HR 0.97, 95%C.I. (0.69, 1.37), *p* = 0.86] (Table 3). The 1-year rejection rate was 3.6% lower (*p* < 0.01) in LRDKT recipients with IgA nephropathy. The 1-year eGFR was similar among both groups of recipients with IgA nephropathy (Table 4).

#### Lupus Nephritis

**FIGURE 4:**
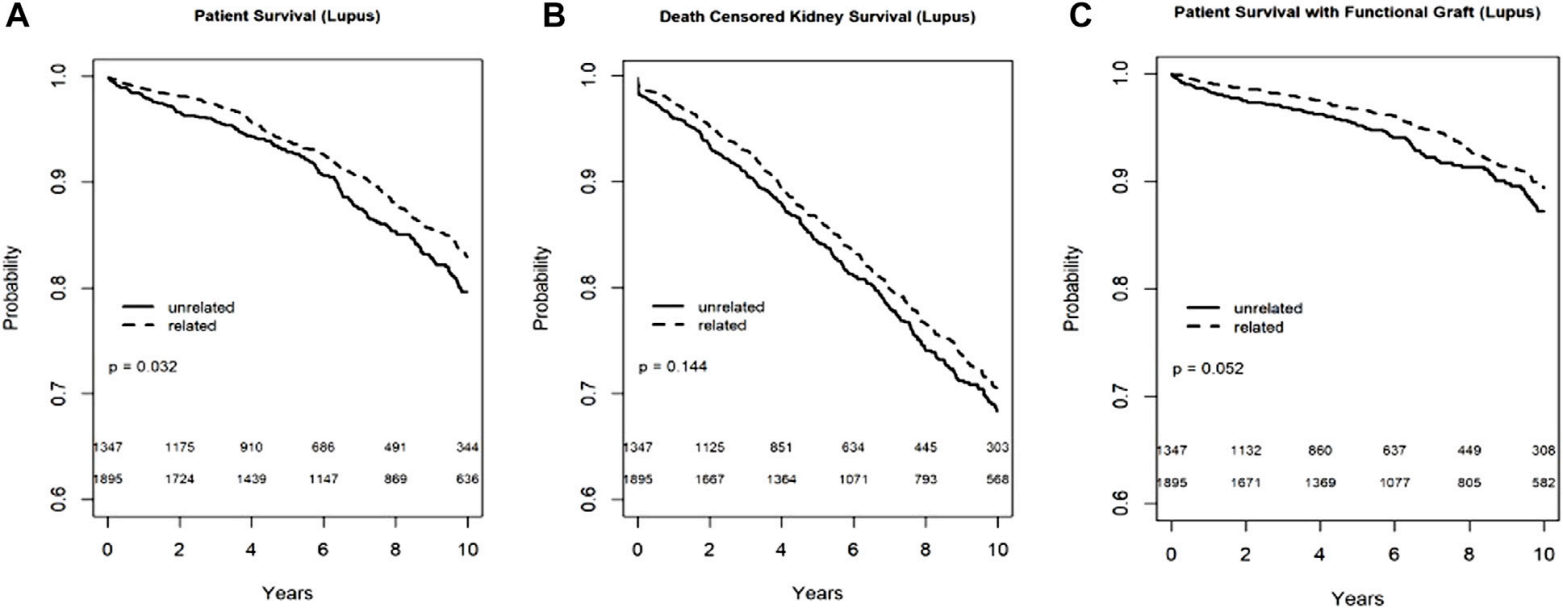
Recipient survival **(A)**, death censored graft survival **(B)** and death with functioning graft **(C)** in patients with lupus nephritis by biologic relationship.

In the Kaplan-Meier analyses for recipient survival and death with functioning graft in those with lupus nephritis, donor-recipient biologic relationship was associated with slightly better recipient survival (log-rank, *p* = 0.03) and death with functioning graft (log-rank *p* = 0.05). However, in the Kaplan-Meier survival for death-censored graft survival in recipients with lupus nephritis, donor-recipient biologic relationship was not associated with worse graft survival (Log-rank, *p* = 0.14) (Figure 4). In the multivariable models, donor recipient relationship was not associated with recipient [HR 0.87, 95%C.I. (0.66, 1.16), *p* = 0.34], death with functioning graft [HR 0.84, 95%C.I. (0.58, 1.23), *p* = 0.38] or death-censored graft survival [HR 0.87, 95%C.I. (0.70, 1.07), *p* = 0.18] (Table 3). The 1-year rejection rate was 2.6% lower (*p* < 0.05) in LRDKT recipients with lupus nephritis. The 1-year eGFR was slightly better in LRDKT recipients with lupus nephritis (Table 4).

#### FSGS

**FIGURE 5:**
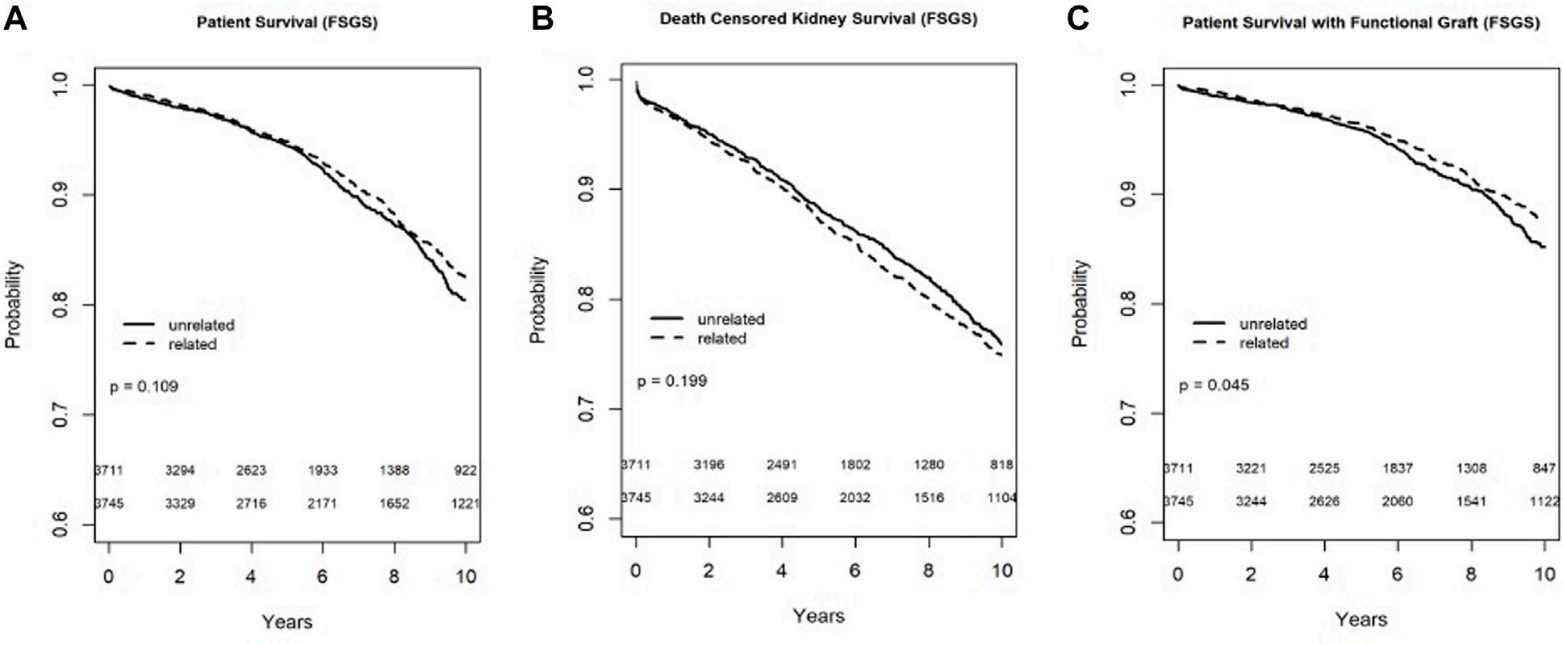
Recipient survival **(A)**, death censored graft survival **(B)**, and death with a functioning graft **(C)** in patients with FSGS by biologic relationship.

In the Kaplan-Meier analysis for recipient survival in those with FSGS, donor-recipient biologic relationship was not a predictor of recipient survival (log-rank, *p* = 0.11). Similarly, in the Kaplan-Meier survival for death-censored graft survival in recipients with FSGS, donor-recipient biologic relationship was not associated with worse graft survival (Log-rank, *p* = 0.19), however death with functioning graft was slightly worse in the FSGS recipients of unrelated living donors (log-rank *p* = 0.04) (Figure 5). In the multivariable models, donor recipient relationship was not associated with recipient [HR 1.06, 95%C.I. (0.87, 1.29), *p* = 0.57], death with functioning graft [HR 0.92, 95%C.I. (0.73, 1.17), *p* = 0.52], or death-censored graft survival [HR 1.09, 95%C.I. (0.93, 1.28), *p* = 0.29] (Table 3). The 1-year rejection rate was 2.1% lower (*p* < 0.01) in LRDKT recipients with FSGS. The 1-year eGFR was slightly better in LRDKT recipients with FSGS (Table 4).

#### Causes of Graft Loss

The causes of graft loss are detailed in Table 5. Unfortunately, due to the large proportion of missing graft loss causes, we were not able to perform a statistical analysis. Seemingly, graft loss due to rejection was less frequently observed in related vs. unrelated recipients in those with membranous, IgA nephropathy and FSGS, while graft loss due to disease recurrence was more frequently observed in the same groups. Recipients with lupus nephritis appear to have similar rates of graft loss and disease recurrence irrespective of the donor-recipient biologic relationship.

**TABLE 5:** Causes of Graft loss in related vs. Unrelated kidney transplant recipients with different GN types N (%).

	Membranous		IgA	
	Unrelated *n* = 750	Related *n* = 959	Unrelated *n* = 3,423	Related *n* = 3,838
Total Graft Loss	197 (26.3)	251 (26.2)	508 (14.8)	714 (18.6)
Acute or Chronic Rejection	81 (41.1)	89 (35.5)	225 (44.3)	269 (37.7)
Recurrent Disease	37 (18.8)	57 (22.7)	64 (12.6)	118 (16.5)
Missing/other	79 (40.1)	105 (41.8)	219 (43.1)	327 (45.8)

	Lupus Nephritis		FSGS	
	Unrelated *n* = 1,347	Related *n* = 1,895	Unrelated *n* = 3,711	Related *n* = 3,745
Total Graft Loss N (%)	325 (24.1)	512 (27.0)	728 (19.6)	862 (23.0)
Acute or Chronic Rejection	167 (51.4)	264 (51.6)	297 (40.8)	320 (37.1)
Recurrent Disease	17 (5.2)	32 (6.2)	128 (17.6)	173 (20.1)
Missing/other	141 (43.4)	216 (42.3)	303 (41.7)	369 (42.9)

Statistical analysis was not possible due to missingness; Total graft loss percentage represents graft loss out of the total number transplant in each GN category; Cause specific graft loss percentage represents cause specific count out of total graft loss count in each GN category.

#### Rejection Rate Among Recipients With Greater Than One HLA Mismatches

In a subgroup analysis of the entire cohort including those with HLA mismatches>1, rejection rate at six- and 12-months were not statistically different in related and unrelated recipients (Supplementary Table S1).

## Discussion

To date our study is the largest analysis with extended follow up of donor-recipient relationship and survival outcomes in adult recipients with different types of glomerular disorders. Our results can be summarized as follows: 1. Donor-recipient biologic relationship was not associated with worse long-term recipient or graft survival in those with membranous, IgA, lupus nephritis or FSGS. 2. Rejection rates were significantly lower in most of the GN recipients of LRDKT. 3. Graft loss due to recurrence was more frequently documented in recipients of related compared to unrelated donor kidney transplants.

There is concern about earlier onset as well as increased risk for GN recurrence among living related kidney transplant recipients (5, 8–11). Multiple studies have shown significant genetic predisposition for GNs including IgA nephropathy (10), FSGS (8), lupus nephritis (9, 11), and membranous (12) nephropathy. The evidence for increased GN recurrence risk among biologically related donor-recipient pair has led many investigators to speculate this effect could be attributed to inherited genetic predisposition for kidney disease (5). Recently, Husain et al. analyzed more than 70,000 living donor kidney transplants (1), of which 22% had GN as the primary cause of ESKD. They found that the donor-recipient biologic relationship was associated with a 5% worse renal allograft survival. Interestingly, the observed association was predominantly noted in kidney transplants from live African American donors, which may be attributed to the higher rates of APOL1 risk variants. Kidneys from donors with high-risk APOL1 variants have been associated (13, 14) with worse allograft survival in the African American population. Therefore, their analysis may not have fully accounted for residual confounding. Nonetheless, due to the large sample size, Husain could detect statistical differences, albeit very small.

Earlier studies in living donor kidney transplant recipients with IgA nephropathy and FSGS had conflicting results. McDonald et al analyzed a small cohort from the ANZDATA registry (15) and reported an 8.5-fold increased risk for IgA recurrence among zero HLA mismatched LDKTx. Nonetheless, graft survival was not different. Similarly, Han et al. in 2010 (16) studied the outcomes of 221 recipients in Korea with IgA nephropathy and noted significantly higher rates of recurrence among living related donor kidney transplant. Interestingly, living related donor was associated with better 10-year graft survival. Taken together, it leads us to reevaluate what constitute the most relevant outcomes of interest and avoid unnecessary impedance to live related donations.

Previous results of studies in recipients with FSGS (17–19) are also inconclusive. A couple of studies showed increased risk for FSGS recurrence and worse graft survival among living related kidney transplant recipients. However, a subsequent study by Kennard et al showed (5) no difference in graft survival despite increased risk for FSGS recurrence among the related donor recipient pair, which is concordant with our graft survival results in our FSGS population.

Kennard et al reported (5) on the outcomes of 2,280 living donor kidney transplants in recipients with primary GN in the ANZDATA registry including IgA, FSGS, MN, and mesangiocapillary GN (MCGN). An increased risk for GN recurrence was noted in living related donor kidney transplant (16.2% LRKTx vs. 10.3% LURTx); especially in recipients with IgA nephropathy (4). However, the 10-year death censored graft survival was similar among living related and unrelated kidney transplants and superior to deceased donor kidney transplant (4).

Outcome data on donor-recipient relationship in lupus nephritis is limited to only two very small studies (20–22) with short-term follow up and conflicting results. Our results indicate significantly lower 1 year rejection rates in recipients of a related donor kidney transplant and comparable 10-year death censored graft survival rate.

An analysis of the European Renal Association-European Dialysis and Transplant Association Registry (23), including over 14,000 primary kidney transplants, suggested similar outcomes with living related vs. unrelated donor kidney transplants in IgA, membranous nephropathy and FSGS. Moreover, organs from living donors outperformed organs from deceased donors except in membranoproliferative glomerulonephritis. Our study complements the above analysis as we included over 19,000 live donor transplants with over 50% first degree related donors compared to only 21% of the entire cohort from the European registry. Reassuringly, the results of the two studies are consistent and both refute the notion of questioning the performance of living related donor transplants in recipients with the studied glomerulonephritis groups. One exception is membranoproliferative, which was not covered in our study due to extensive heterogeneity of this disorder.

Our study results are validated by findings of Kennard (5), Han (16), and others (4, 23). We complement their reports by expanding our study to include other types of GN, the largest number of living donor recipients with a long follow up. Taken together, the data support the value of living related donor kidney transplant in recipients with GN despite the perceived increased risk for recurrence.

### Strengths and Limitations

Our study has several strengths and limitations. This study is the largest cohort of living donor transplant in recipients with GN including 19,668 recipient-donor pairs. Biologic relationship between donor recipient pairs was clearly defined in SRTR and restricted to parent, sibling, twin, or child. One limitation is that some GN disorders, more than others, can be primary or secondary in nature, which is not clearly defined in the SRTR standard analysis file. Another limitation is that a cause specific graft loss was either listed as missing or “other” in a large portion of our cohort. This limitation precluded formal analyses of the association between donor-recipient biologic relationship and disease recurrence. The lack of specific data on the cause of graft loss in a substantial proportion of kidney transplants could be due to different center practices and different thresholds to perform kidney transplant biopsies. Nonetheless, death and graft loss are strictly tracked in the SRTR allowing us to complete a robust analysis to help settle this important question.

### Conclusion

In this large cohort study, biologic relationship was associated with lower rejection rates in IgA, lupus nephritis, and FSGS. Additionally, it was not associated with worse recipient or graft survival in any of the studied GN groups: MN, IgA, lupus nephritis, or FSGS. These findings are consistent with the known benefits of living related donor kidney transplant and counters reports about the adverse impact of donor recipient biologic relationship on allograft outcomes.

## Data Availability

The original contributions presented in the study are included in the article/Supplementary Material, further inquiries can be directed to the corresponding author.

## Abbreviations

CI, confidence interval; DCGS, death censored graft survival; ESKD, end stage kidney disease; FSGS, focal segmental glomerulosclerosis; GN, glomerulonephritis; HLA, human leukocyte antigen; HRSA, the health resources and services administration; KDIGO, kidney disease improving global outcomes; KTA, kidney alone transplant; LDKTx, living donor kidney transplant; LRKTx, living related donor kidney transplant; LUKTx, living unrelated donor kidney transplant; MN, membranous nephropathy; OPTN, organ procurement and transplantation network; SPK, simultaneous pancreas-kidney; SRTR, scientific registry of transplant recipients; US, United States; UNOS, united network for organ sharing.
