# Supplementary material for: Living Donor Kidney Transplant in Recipients With Glomerulonephritis: Donor Recipient Biologic Relationship and Allograft Outcomes
Source: Transpl Int. 2023 May 5;36:11068. doi: 10.3389/ti.2023.11068 (PMC10195883; doi:10.3389/ti.2023.11068)
Supplement: Supplementary file 1 [file Table1.pdf]

Table S1. Rejection Rates by Recipient-Donor Relationship among Recipients with HLA Mismatches > 1 N (%)

	Unrelated N=3711	Related N=3745	p-value
HLA MM>1	3602 (97)	2773 (74)	
6-Month Rejection	258 (8.8)	168 (8.3)	0.56
12-Month Rejection	346 (12.2)	228 (11.6)	0.58

HLA: Human Leukocyte Antigen; MM: Mismatch
